# Gut microbiome and metabolome to discover pathogenic bacteria and probiotics in ankylosing spondylitis

**DOI:** 10.3389/fimmu.2024.1369116

**Published:** 2024-04-22

**Authors:** Yupeng Lai, Wenli Tang, Xiao Luo, Huihui Zheng, Yanpeng Zhang, Meiying Wang, Guangchuang Yu, Min Yang

**Affiliations:** ^1^ Department of Rheumatology and Immunology, Nanfang Hospital, Southern Medical University, Guangzhou, China; ^2^ Department of Rheumatology and Immunology, The First Affiliated Hospital of Shenzhen University, Shenzhen Second People’s Hospital, Shenzhen, China; ^3^ Department of Bioinformatics, School of Basic Medical Sciences, Southern Medical University, Guangzhou, China; ^4^ Department of Pharmacy, Nanfang Hospital, Southern Medical University, Guangzhou, China; ^5^ Department of Laboratory, The First Affiliated Hospital of Shenzhen University, Shenzhen Second People’s Hospital, Shenzhen, China

**Keywords:** microbiome, metabolome, ankylosing spondylitis, *Ruminococcus gnavus*, biotin, CaCo-2, *Bacteroides uniformis*, oxypurinol

## Abstract

**Objective:**

Previous research has partially revealed distinct gut microbiota in ankylosing spondylitis (AS). In this study, we performed non-targeted fecal metabolomics in AS in order to discover the microbiome–metabolome interface in AS. Based on prospective cohort studies, we further explored the impact of the tumor necrosis factor inhibitor (TNFi) on the gut microbiota and metabolites in AS.

**Methods:**

To further understand the gut microbiota and metabolites in AS, along with the influence of TNFi, we initiated a prospective cohort study. Fecal samples were collected from 29 patients with AS before and after TNFi therapy and 31 healthy controls. Metagenomic and metabolomic experiments were performed on the fecal samples; moreover, validation experiments were conducted based on the association between the microbiota and metabolites.

**Results:**

A total of 7,703 species were annotated using the metagenomic sequencing system and by profiling the microbial community taxonomic composition, while 50,046 metabolites were identified using metabolite profiling. Differential microbials and metabolites were discovered between patients with AS and healthy controls. Moreover, TNFi was confirmed to partially restore the gut microbiota and the metabolites. Multi-omics analysis of the microbiota and metabolites was performed to determine the associations between the differential microbes and metabolites, identifying compounds such as oxypurinol and biotin, which were correlated with the inhibition of the pathogenic bacteria *Ruminococcus gnavus* and the promotion of the probiotic bacteria *Bacteroides uniformis*. Through experimental studies, the relationship between microbes and metabolites was further confirmed, and the impact of these two types of microbes on the enterocytes and the inflammatory cytokine interleukin-18 (IL-18) was explored.

**Conclusion:**

In summary, multi-omics exploration elucidated the impact of TNFi on the gut microbiota and metabolites and proposed a novel therapeutic perspective: supplementation of compounds to inhibit potential pathogenic bacteria and to promote potential probiotics, therefore controlling inflammation in AS.

## Introduction

Ankylosing spondylitis (AS) is an inflammatory disease that affects the axial spine, peripheral joints, and entheses. In previous years, the diagnosis of AS has relied on the 1984 modified New York criteria, which required radiographic changes in the sacroiliac joint for diagnosis (1). In recent years, the Assessment of SpondyloArthritis International Society (ASAS) introduced the axial spondyloarthritis (axSpA) classification criteria in 2009 to encompass both non-radiographic and radiographic axSpA (also known as AS) (2). In this report, AS was diagnosed according to the 1984 criteria or the 2009 criteria.

The pathogenesis of AS was possibly associated with the microbiota due to differences in the microbiota between patients with AS and healthy controls being found (3). Using 16S ribosomal RNA amplicon-based profiles, researchers found that, at the family level, there were more Lachnospiraceae in patients with AS, but fewer Prevotellaceae and Paraprevotellaceae in AS cases compared with healthy controls (4). A recent study using shotgun metagenomic sequencing further confirmed that seven bacterial species were identified to be differentially abundant between AS cases and healthy controls (5). Commensal gut microbes, as demonstrated in a mouse study, broadly influence metabolites, thereby modifying the host-generated signaling molecules (6). However, the impact of the interaction between microbes and metabolites on AS has seldom been explored. To our knowledge, only one targeted analysis of metabolites that displayed a decreased super pathway of l-tryptophan, which is connected with the metagenome, has been reported (7). A study on healthy fecal samples revealed that an untargeted approach provides a better correlation with the microbiome data than does targeted metabolomics. For instance, targeted analysis misidentifies 30% of the targeted data and could lead to incorrect interpretations (8). In AS, conducting untargeted metabolomics on fecal samples and exploring correlations with the microbiome offer the opportunity to accurately reveal the biological mechanisms between metabolites and the microbiota, providing valuable data for understanding the pathogenesis of AS.

Treatment with tumor necrosis factor inhibitor (TNFi) is a prevalent therapy for AS worldwide, and continuing TNFi is recommended by the 2019 ACR Network to achieve stable disease activity (9). Since TNFi is an important therapy for AS, its effect on the microbiota and metabolome is of interest. A cross-sectional study compared the differences in the microbiota among TNFi-untreated AS cases, TNFi-treated AS cases, and healthy controls (5). As is known, cross-sectional studies could not elucidate a causal relationship. It is therefore necessary to conduct prospective cohort studies in order to compare the effects of TNFi on the microbiome and metabolome before and after treatment in AS.

Both microbes and metabolites act directly on intestinal cells, and they are likely to induce intestinal inflammation. This was confirmed by a study that found that up to 60% of patients with spondyloarthritis (SpA) demonstrated subclinical gut inflammation, with up to 10% of those progressing to a clinically overt Crohn’s disease, which is a type of inflammatory bowel disease (IBD) (10). IBD and AS share common clinical manifestations, indicating a closer relationship between the gut and the joints in their development (11). The discovery of perturbations of the microbiome–metabolome interface in IBD identified several potential diagnostic and therapeutic targets (12). Thus, it is possible to display the association between the microbiome and metabolome in AS and to perform validation experiments for these relationships.

In this work, we conducted a prospective, longitudinal cohort study to collect stool samples from 29 patients with AS before TNFi therapy (named the pre-treatment group) and after TNFi therapy for 1 year (named the post-treatment group). The research findings are elaborated through five sections: 1) differences in the microbiome between patients with AS and healthy controls and TNFi on differential microbes; 2) differences in the metabolome between patients with AS and healthy controls and TNFi on differential metabolites; 3) putative association between the microbes and metabolites; 4) validation experiments for the microbes and metabolites; and 5) validation experiments for the microbes on enterocytes.

## Materials and methods

### Subject recruitment

We conducted a prospective, longitudinal cohort study including patients with AS who did not have any previous TNFi therapy. When these patients were hospitalized, we collected their clinical indicators and stool samples. After a 12-month TNFi therapy, the patients were requested to go back to the hospital for an examination and to donate stool samples. After collection, the stool samples were frozen and stored at −80°C. A total of 29 patients with AS and 31 healthy controls were included. The criteria were designed to avoid the impact of other diseases, drugs, or dietary habits on the microbiota and metabolites. The detailed inclusion and exclusion criteria for patients and healthy controls are provided in the **Supplementary Material**. This study was approved by the Ethics Committee of Shenzhen Second People’s Hospital, with approval ID 20210620213357028. Written informed consent was obtained from all participants, including both patients and controls, prior to their involvement in the studies.

### DNA extraction and metagenomic sequencing

The DNAs extracted from the stool samples of 29 AS cases pre-treatment, 29 AS cases post-treatment, and 31 healthy controls were sequenced using the BGI sequencing platform (BGI, Shenzhen, China) with 300- to 400-bp DNA fragment libraries. The original sequencing data were filtered using SOAPnuke v2.2.1 to obtain clean data. The T2T-CHM13 reference (Telomere-to-Telomere Consortium Human Genome Assembly in Chromosome 13) was utilized and the Bowtie 2 v2.3.5.1 tool was employed to eliminate host contaminant sequences. Subsequently, microbial functional annotation was performed using HUMAnN3 v3.0.1. Finally, according to the bacterial library in Kraken2 v2.0.8-beta, Kraken2 and Bracken were employed for species annotation.

### Metabolite profiling from stool samples

For this experiment, we used the Waters UPLC I-Class Plus system (Waters, Milford, MA, USA) coupled with the Q Exactive high-resolution mass spectrometer (Thermo Fisher Scientific, Waltham, MA, USA) for the separation and detection of metabolites. After transferring the off-line mass spectrometry data to the Compound Discoverer 3.3 software (Thermo Fisher Scientific, Waltham, MA, USA) and conducting analysis on the mass spectrometry data in conjunction with BMDB (BGI metabolome database), the mzCloud database, and the online ChemSpider database, a data matrix was generated. This matrix contained details such as the metabolite peak area and the identification outcomes. The detailed experiment on metabolite profiling is presented in the **Supplementary Material**.

### Effects of metabolites on species growth

*Ruminococcus gnavus* was grown in an anaerobic culture bag (Mitsubishi, Tokyo, Japan) in a brain–heart infusion (BHI) broth supplemented with yeast extract and hemin (BHI-YH) [37 g/L BHI (HKM, Guangdong, China) supplemented with 5 g/L yeast extract (Coolaber, Beijing, China) and 5 mg/L hemin (Sigma-Aldrich, St. Louis, MO, USA)], as previously described (13). *Bacteroides uniformis* was grown in an anaerobic culture bag (Mitsubishi, Tokyo, Japan) in 37 g/L BHI (HKM, Guangdong, China) supplemented with 0.4 g/L l-cysteine (Dogesce, Beijing, China), 0.01 g/L hemin (Keyinbio, Lexington, KY, USA), and 0.5 mg/L vitamin K3 (Goldwheat, Guangdong, China) (14, 15). Bacteria in the logarithmic growth phase were used for the experiment (OD_600_ = 0.6). Analysis on the microbiome and metabolome found that *R. gnavus* was negatively associated with the metabolite oxypurinol. Oxypurinol, at different concentrations ranging from 320 mg/L to 2.5 mg/L, was added to *R. gnavus*, and the OD_600_ was recorded every 8 h. Analysis of the microbiome and metabolome found that *B. uniformis* was positively associated with the metabolite biotin. Biotin, at different concentrations ranging from 5,000 mg/L to 0.0005 mg/L, was added to *B. uniformis*, and the OD_600_ was recorded every 8 h.

### Interaction between species and Caco-2 cells

Enterocytes are the most abundant cells in the intestine. The Caco-2 cell line (ATCC, Manassas, VA, USA) was used to mimic the enterocytes in this work in order to study the interactions between species and Caco-2 cells. The Caco-2 cells were cultured in a T-25 culture flask with 5 mL of complete culture medium, which consisted of 86% minimum essential medium (Procell, Wuhan, China), 10% fetal bovine serum (FBS; NEWZERUM, Christchurch, New Zealand), 1% GlutaMAX, 1% non-essential amino acids, 1% sodium pyruvate (all from Procell, Wuhan, China), and 1% penicillin and streptomycin (Gibco, Carlsbad, CA, USA). The Caco-2 cells were seeded at 2.5 × 10^5^ cells/well onto 24-well tissue culture plates with 0.5 mL complete culture medium and 10% FBS. After 24 or 36 h, the previous medium in the wells was discarded and 10% supernatant of the species was added to complete the culture medium to co-culture with the cells, while the control group received only the complete culture medium. In the healthy model, to represent a healthy intestine, 10% supernatant of *R. gnavus* and 10% supernatant of *B. uniformis* were added to the Caco-2 cells. To further mimic the inflammatory gut environment in patients with axSpA, dextran sodium sulfate (DSS) was used to induce inflammatory Caco-2 cells. In the 5% DSS-induced inflammatory model, 5% supernatant of *R. gnavus* and 5% supernatant of *B. uniformis* were added to the Caco-2 cells. A cell counting kit-8 (CCK-8; Selleck, Shanghai, China) assay was performed to evaluate the proliferation affected by the supernatants of *R. gnavus* and *B. uniformis*. After extraction of the total RNA using TRIzol (Vazyme, Nanjing, China), quantitative real-time reverse transcription polymerase chain reaction (qRT-PCR; Yeasen, Shanghai, China) was performed to examine the effect of *R. gnavus* and *B. uniformis* on the Caco-2 cells’ mRNA expression of interleukin-18 (IL-18). Enzyme-linked immunosorbent assay (ELISA; Elabscience, Wuhan, China) was performed to display the effect of *R. gnavus* and *B. uniformis* on the IL-18 protein of Caco-2 cells.

### Microbiome statistical analysis

To obtain the relative abundance of bacteria, the abundance of the different bacterial species was divided by the total bacterial count in each sample. Principal coordinates analysis (PCoA) based on the Bray–Curtis distance and permutational multivariate analysis of variance (PERMANOVA) were used to compare the microbial profiles of the three groups (i.e., the before group, the control group, and the after group). The Bray–Curtis distance is a distance metric used to calculate community similarity, which considers the feature abundance data in the calculation. To determine the differentially abundant species between two groups, the Wilcoxon rank-sum test and the false discovery rate (FDR) were calculated, and an adjusted *p*-value <0.05 was used as the standard. Only those microbial species with a relative abundance value above 0.001% in at least 10% of the samples were considered.

### Metabolome statistical analysis

PCoA based on the Bray–Curtis distance and PERMANOVA were used to compare the metabolite profiles among the three groups. All measured metabolites (which were first log2-transformed) were taken into consideration. Orthogonal projections to latent structures discriminant analysis (OPLS-DA) was conducted between two groups to obtain the variable importance in projection (VIP) score for each metabolite, which represents their contribution to group discrimination. A relevant model validation was conducted by performing 200 response permutation tests. The differential compounds between two groups were selected using the following criteria: compounds with VIP values ≥1 from the OPLS-DA, log2 fold change (LFC) ≥1.2 or ≤0.83, and a *p* < 0.05 from the Wilcoxon rank-sum test.

### Multi-omics statistical analysis

Spearman’s correlation analysis was conducted to evaluate the relationship between the differentially abundant species and the metabolites in the pre-treatment group compared with the healthy control group. The *p*-values were adjusted using the FDR approach, and those with an adjusted *p* < 0.05 were considered to be statistically significant.

### Experiment statistical analysis

The *t*-test and analysis of variance (ANOVA) were used for the analysis of the bacterial experiments and the cell experiments.

## Results

### Overview of the demographic characteristics

A total of 29 patients and 31 healthy controls were included in this study, with the 29 patients receiving a 1-year follow-up. Stool samples were collected from patients with AS before TNFi treatment [the pre-treatment (Pre) group], after TNFi treatment [the post-treatment (Post) group], and from healthy controls (the HC group). All of the stool samples were examined for metagenome and metabolome. The demographics of the patients (pre-treatment) and the HCs displayed no differences in age and gender (**Supplementary Table S1**). A total of 29 patients received TNFi treatment (adalimumab, *n* = 27; etanercept, *n* = 2) for 1 year. Patients with AS received standard doses of TNFi treatment in the first 6 months, followed by a 50% dose reduction in the subsequent 6 months. This tapering regimen followed the tapering protocol from a cohort study conducted in South Korea (16). This study employed three recognized scoring methods to assess the impact of TNFi treatment on the disease activity of AS. These three scoring methods were as follows: the Ankylosing Spondylitis Disease Activity Score (ASDAS) (17), the Bath Ankylosing Spondylitis Disease Activity Index (BASDAI) (18), and the Bath Ankylosing Spondylitis Functional Index (BASFI) (19). The paired-samples *t*-tests for the patients with AS in the Pre and Post groups showed that the BASDAI, ASDAS, and BASFI significantly decreased in the Post group compared with the Pre group (*p* < 0.05), and all indices achieved low disease activity (**Supplementary Table S2**).

### The microbiome

**Figure 1:**
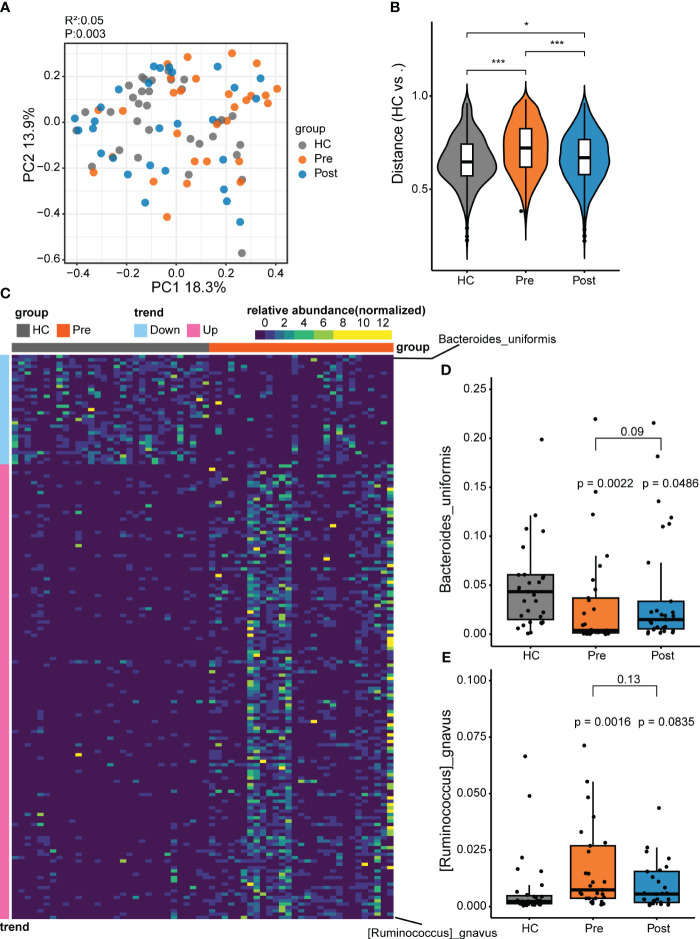
Differential microbes between patients with ankylosing spondylitis (AS) and healthy controls (HCs). **(A)** The microbial composition patterns were analyzed using principal coordinates analysis (PCoA). **(B)** Comparison of the relative abundance using the Bray–Curtis distance between samples in the HC group, pre-treatment AS group (*Pre*), and post-treatment AS group (*Post*). *p < 0.05, ***p < 0.001. **(C)** Relative abundance for species comparison between the Pre and HC groups to identify the depleted and enriched species in AS. **(D)** *Bacteroides uniformis* nearly recovered to the HC level with tumor necrosis factor inhibitor (TNFi) therapy. **(E)** *Ruminococcus gnavus* recovered to the HC level with TNFi therapy.

By using the metagenomic sequencing system and by profiling the microbial community taxonomic composition, we annotated 7,703 species. The microbial composition patterns were analyzed using PCoA based on the Bray–Curtis distance and PERMANOVA. The overall composition of the gut microbiota varied significantly among the control group, the pre-treatment group, and the post-treatment group, with the post-treatment group showing a closer alignment with the control group (**Figure 1A**). The distances between the samples displayed that the difference between the Pre and HC groups was greater than that between the Post and HC groups (**Figure 1B**), suggesting microbial recovery from the pathological pattern of the pre-treatment group. This implies that TNFi therapy had an impact on the gut microbiota.

To further explore the microbial patterns in the three groups, the Wilcoxon rank-sum test and the FDR were performed. Comparison of the Pre group (i.e., the AS patient group) and the HC group revealed 170 featured bacterial species that showed significant differences (adjusted *p* < 0.05) between patients with AS and healthy controls (**Figure 1C**; **Supplementary Figure S1**). Only two differential species between the Post and HC groups and six differential species between the Post and Pre groups were found (**Supplementary Figure S2**). Of the 170 significantly different species compared with the HC group, 137 displayed more abundance and 33 displayed lower abundance (**Supplementary Figure S1**). The 137 enriched microbes of AS comprised mostly the phylum Firmicutes (consisting of the genera *Clostridium*, *Ruminococcus*, *Faecalibacterium*, *Anaerostipes*, *Anaerotruncus*, and *Blautia*), with a small contribution of the phyla Pseudomonadota and Actinomycetota. In contrast, most of the 33 depleted species in patients with AS were in the phylum Bacteroidetes, including the *Bacteroides*, *Parabacteroides*, *Phocaeicola*, and *Alistipes* genera. In the Pre group (i.e., the AS patient group), the enriched bacteria might be pathogenic microbes related to the disease. In the Pre group, compared with the HC group, *R. gnavus* ranked first in the enriched bacteria. *R. gnavus* has often been reported to be elevated in patients with various immune diseases, including IBD, AS, and systemic lupus erythematosus (5, 20, 21). It is a possible pathogenic bacterium associated with immune diseases. Compared with the HC group, the predominant depleted bacteria belong to the phylum Bacteroidetes, most of which have the capacity to produce butyrate and have been recognized as potential probiotics for future development (22). Among the Bacteroidetes species, *B. uniformis* ranked first, being a butyrate-producing strain and a potential probiotic. Therefore, to further understand these two unique species, we conducted comparative analyses between the Pre, Post, and HC groups. The increase of *R. gnavus* in patients returned to a level similar to that in the HC group after treatment. Similarly, the decrease of *B. uniformis* in patients increased to a level similar to that in the HC group after treatment (**Figures 1D, E**).

### The metabolome

**Figure 2:**
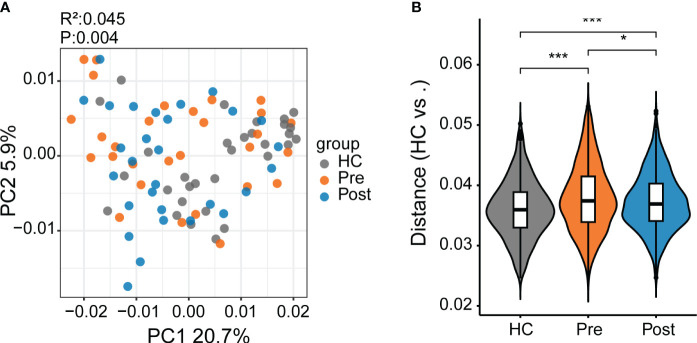
Differential metabolites between patients with ankylosing spondylitis (AS) and healthy controls (HCs). **(A)** The metabolite composition patterns were analyzed using principal coordinates analysis (PCoA). **(B)** Comparison of the metabolite differences using the Bray–Curtis distance between the samples in the HC group, the pre-treatment AS group (*Pre*), and the post-treatment AS group (*Post*). *p < 0.05, ***p < 0.001.

**Figure 3:**
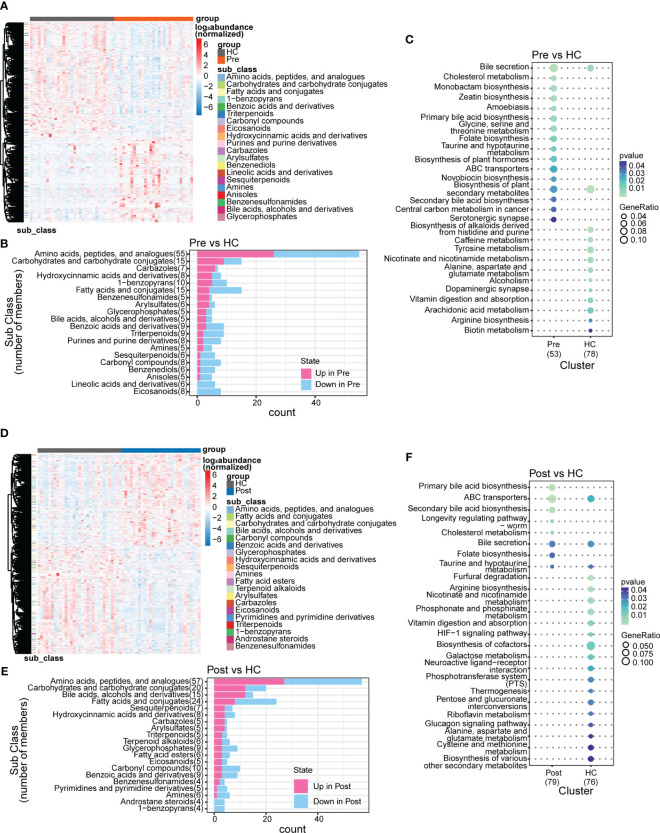
Subclasses and enriched pathways of the differential metabolites between the ankylosing spondylitis (AS) patients before treatment (*Pre*), the AS patients after treatment (*Post*), and the healthy controls (HCs). **(A)** Top 20 subclasses of the differential metabolites between the Pre and HC groups displayed as heatmaps. **(B)** Details of the top 20 subclasses of differential metabolites between the Pre and HC groups displayed as bar charts. **(C)** Top 20 enriched pathways of the differential metabolites between the Pre and HC groups, with the GeneRatio representing the ratio of input compounds. **(D)** Top 20 subclasses of the differential metabolites between the Post and HC groups displayed as heatmaps. **(E)** Details of the top 20 subclasses of differential metabolites between the Post and HC groups displayed as bar charts. **(F)** Top 20 enriched pathways of the differential metabolites between the Post and HC groups, with the GeneRatio representing the ratio of input compounds.

In this study, we identified 50,046 metabolites and compared their major distribution patterns in the three groups using PCoA and PERMANOVA. The Pre group was separated from the HC group, while the Post group resembled both the Pre group and the HC group, presenting similar distributions to the microbiome, which implies a recovery from the disease state (**Figure 2A**). The Bray–Curtis distance displayed that the difference between the Pre and HC groups was similar to that between the Post and HC groups (**Figure 2B**). To further discriminate the differential metabolites, OPLS-DA was performed (**Supplementary Figures S3A–C**), and relevant model validity was verified by performing 200 response permutation tests (**Supplementary Figures S3D–F**). The OPLS-DA model was found valid as the Q2 regression showed a negative intercept (23). The differential metabolites were selected based on the following criteria: 1) VIP ≥ 1 from the OPLS-DA model; 2) a fold change ≥1.2 or ≤0.83; and 3) a *p* < 0.05. There were 1,166 differential metabolites between the Pre group and the HC group, 334 between the Pre group and the Post group, and 1,131 between the Post group and the HC group. **Figure 3A** displays the differential metabolites between the Pre group and the HC group, while **Figure 3B** presents a summary of the top 20 subclasses [the chemical taxonomy in the HMDB database (24)] of metabolite features. These 20 metabolites are the ones with the greatest differences and the largest number between the Pre group and the HC group. Moreover, the patterns of the subclasses between the Post group and the HC group (**Figures 3D, E**) and those between the Post group and the Pre group are presented (**Supplementary Figures S4A, B**). These revealed the different metabolite features between groups. Compared with the HC group, TNFi therapy did not change most of the top 20 differential metabolites in the Pre group. The differential metabolites—such as amino acids, peptides, and analogues; carbohydrates and carbohydrate conjugates; carbazoles, hydroxycinnamic acids, and derivatives; 1-benzopyrans, fatty acids, and conjugates; benzenesulfonamides, arylsulfates, glycerophosphates, bile acids, alcohols, and derivatives; benzoic acids and derivatives; and triterpenoids, amines, sesquiterpenoids, and carbonyl compounds—remained the same in the Post–HC comparison group as that in the Pre–HC comparison group. Only four subclasses of differential metabolites, which included purines and purine derivatives, benzenediols, anisoles, and linoleic acids and derivatives, changed after TNFi therapy. To further analyze the Kyoto Encyclopedia of Genes and Genomes (KEGG) (25) metabolic pathways which the differential metabolites were enriched in, clusterProfiler (26) was utilized to determine the enriched metabolic pathways, thereby contributing to the interpretation of the biological phenotypes. In this study, the metabolic pathways with *p* < 0.05 were identified as significantly enriched pathways for the differential metabolites. Subsequently, the top 20 enriched pathways exhibiting the smallest *p*-values were graphically represented using bubble charts to display the differential metabolites between the Pre group and the HC group, between the Post group and the HC group, and between the Post group and the Pre group (**Figures 3C, F**; **Supplementary Figure S4C**). The GeneRatio represents the ratio of input compounds that were annotated in the enriched pathways in the bubble charts (26). The enrichment pathways in the Pre group were different from those in the HC group. The enriched pathways in the Pre group mostly comprised bile-related metabolism pathways, such as bile secretion, cholesterol metabolism, primary bile acid biosynthesis, taurine and hypotaurine metabolism, and secondary bile acid biosynthesis. The enriched pathways in the HC group comprised purine metabolism, vitamin digestion and absorption, biotin metabolism, and metabolism of various amino acids. We also evaluated the change of the enriched pathways in the Pre group after TNFi therapy. Compared with the HC group, several enriched pathways in the Pre group were changed after TNFi therapy, such as monobactam biosynthesis, zeatin biosynthesis, amoebiasis, glycine, serine and threonine metabolism, plant hormone biosynthesis, novobiocin biosynthesis, plant secondary metabolite biosynthesis, central carbon metabolism in cancer, and serotonergic synapse. A few enriched pathways in the Pre group, such as bile secretion, cholesterol metabolism, primary bile acid biosynthesis, folate biosynthesis, ABC transporters, and secondary bile acid biosynthesis, remained similar in the Post–HC comparison group to that in the Pre–HC comparison group. The enriched pathways in the HC group remained nearly the same in both the Pre–HC and Post–HC comparison groups, such as vitamin digestion and absorption and the metabolism of various amino acids.

### The putative mechanistic associations between microbes and metabolites

**Figure 4:**
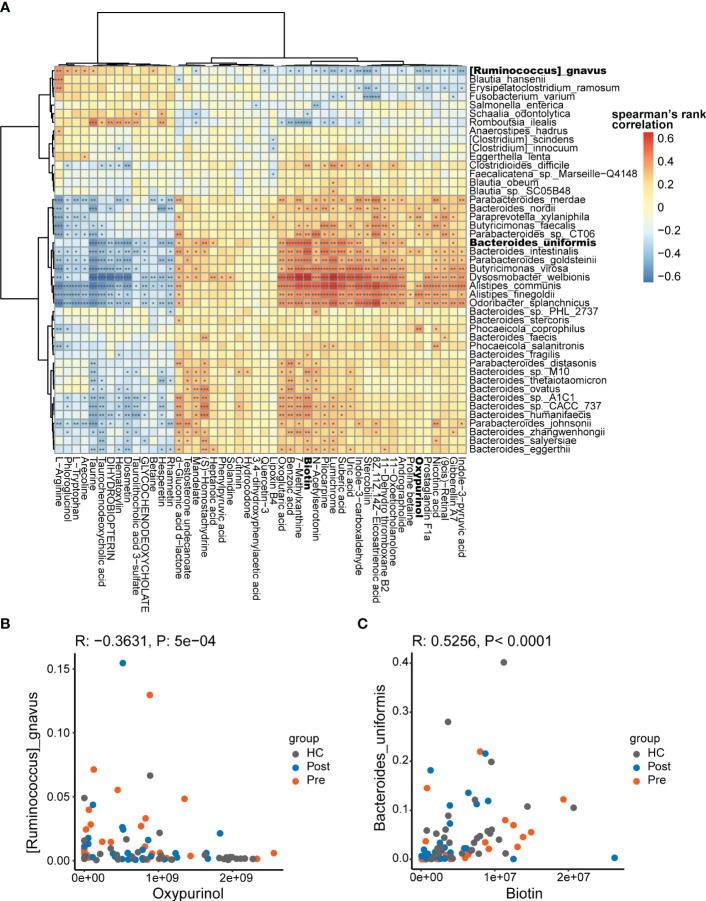
Potential mechanistic associations between microbes and metabolites. **(A)** Spearman’s correlations between the differentially abundant species and the differentially abundant metabolites. **(B)** *Ruminococcus gnavus* was in negative association with oxypurinol. **(C)** *Bacteroides uniformis* was in positive association with biotin *p < 0.05, **p < 0.01, and ***p < 0.001.

The multi-omics nature of this dataset provided an excellent opportunity to discover the association between microbes and metabolites. Spearman’s correlations were conducted for the differentially abundant species and metabolites, focusing on the Pre and HC groups. Consequently, 1,003 associations between the species and the metabolites (**Figure 4A**) were found to be significantly different (*p* < 0.05).

The correlations between the differential species and metabolites showed both positive and negative correlations. The positive correlations suggest that differential species might produce specific differential metabolites or that specific differential metabolites might promote the proliferation of specific differential species. The negative correlations suggest that differential species might consume specific differential metabolites or that specific differential metabolites might inhibit the proliferation of specific differential species. In the correlation analysis, *R. gnavus* was found to be significantly associated with 34 metabolites (*p* < 0.05). In the negative association with *R. gnavus*, oxypurinol ranked fifth based on the correlation coefficients (**Figure 4B**). Oxypurinol is an isostere of xanthine, both having the molecular formula C_5_H_4_N_4_O_2_. More interestingly, oxypurinol has been reported in previous studies to suppress the phylum Firmicutes, especially the genus *Ruminococcus* (27). *R. gnavus* belongs to the genus *Ruminococcus* within the phylum Firmicutes Thus, oxypurinol is considered a potential inhibitor of *R. gnavus*, which was verified in subsequent experiments. Similarly, we found that *B. uniformis* was correlated with 41 metabolites, in which biotin ranked first in the positive association (**Figure 4C**). Biotin, also known as vitamin H or vitamin B7, is a nutrient essential for the growth of various bacteria. It serves as a crucial substrate for butyrate-producing bacteria, which commonly rely on biotin for optimal growth. In the absence of biotin, some bacteria fail to reach their optimal growth state (28). *B. uniformis* is also a butyrate-producing bacterium (29). Supplementation with biotin could potentially promote the growth of *B. uniformis*. Thus, biotin was verified as the promoter of *B. uniformis* in subsequent experiments.

### The effect of metabolites on species

**Figure 5:**
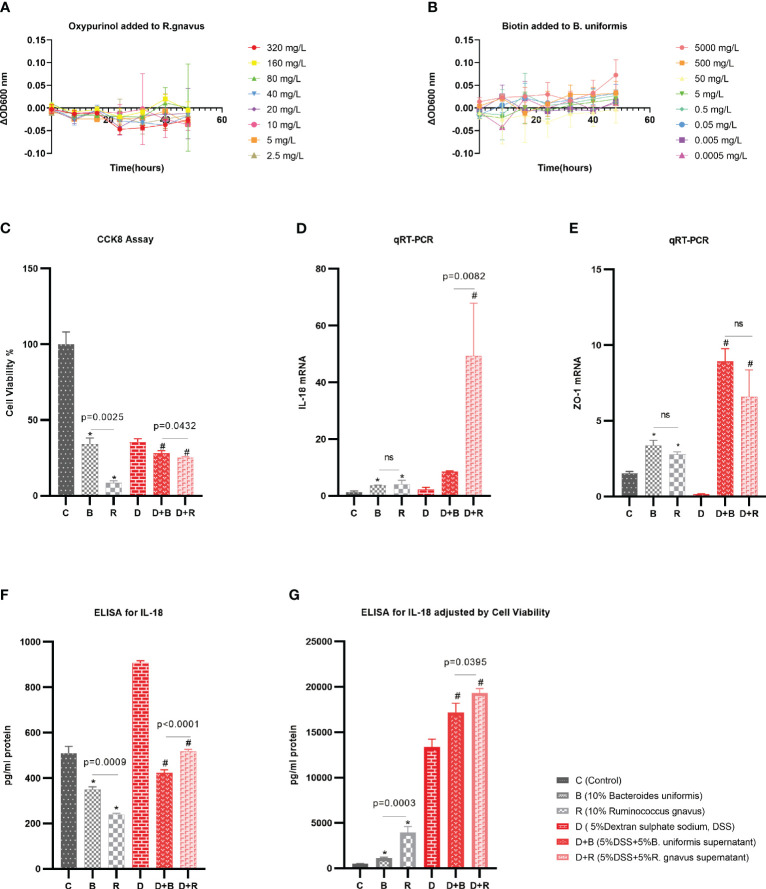
Validation experiments for the effects of the metabolites on the species and the effects of the species on the enterocytes. **(A)** The supernatant of oxypurinol impacted *Ruminococcus gnavus* proliferation. **(B)** The supernatant of biotin impacted *Bacteroides uniformis* proliferation. **(C–F)** Effects of the supernatants from *R. gnavus* and *B*. *uniformis* on the proliferation of the enterocyte cell model, the Caco-2 cell line, for 36 h **(C)**; on the IL-18 mRNA expression of the Caco-2 cells for 36 h **(D)**; on the tight junction protein ZO-1 mRNA expression of Caco-2 cells for 36 h **(E)**; and on the IL-18 protein expression by ELISA for 36 h **(F)**. **(G)** Results of the IL-18 protein expression adjusted by cell viability and qRT-PCR *p<0.05 in health model, ^#^ *p*<0.05 in inflammatory model, ns, non significance.

Based on the previous Spearman’s correlation analysis, validation experiments were performed. Oxypurinol was verified to reduce the abundance of Firmicutes (with *Ruminococcus* included at the species level) with a concentration of 40 mg/L (27). Biotin was previously reported to support the growth of human butyrate-producing gut bacteria (28), and 75 g/L biotin was found to improve the gut microbiota of mice (30). *B. uniformis* is one of the butyrate-producing species (31). Thus, experiments were performed to demonstrate that oxypurinol inhibits the growth of *R. gnavus* and that biotin promotes the growth of *B. uniformis*. The optical density (OD) at 600 nm was used to evaluate the growth of species. Considering that dimethylsulfoxide (DMSO) might influence the growth of species, a change in OD_600_ [calculated as ΔOD_600_ = (metabolite + DMSO + culture medium + bacteria)OD_600_ − (DMSO + culture medium + bacteria)OD_600_] was used to examine the effect of metabolites on the growth of species. As expected, oxypurinol significantly inhibited the growth of *R. gnavus* (*p* < 0.05) at concentrations of 320, 40, 20, 10, 5, and 2.5 mg/L (**Figure 5A**). Similarly, biotin significantly promoted the growth of *B. uniformis* (*p* < 0.05) at concentrations of 5,000 and 500 mg/L (**Figure 5B**). The results of these experiments suggest that oxypurinol suppresses the growth of *R. gnavus*, while a high concentration of biotin promotes the growth of *B. uniformis*.

### The effect of species on enterocytes

The Caco-2 cell line, which morphologically resembles enterocytes, is commonly used as a cell model of enterocytes in pharmacology research (32). In this work, the effect of the species supernatants on Caco-2 cells was displayed by determining the cell viability, mRNA, and ELISA after the addition of these supernatants to the Caco-2 cells for 36 h. In both the healthy model and the 5% DSS-induced inflammatory model, the cell viability as evaluated using the CCK-8 assay showed that the supernatant of *R. gnavus* inhibited the growth of Caco-2 cells compared with that of *B. uniformis* (**Figure 5C**). In both the healthy model and the 5% DSS-induced inflammatory model, the qRT-PCR normalized by the ΔΔCT analysis displayed that *R. gnavus* induced more inflammatory IL-18 mRNAs than did *B. uniformis* (**Figure 5D**), while the induction of the tight junction protein ZO-1 by *R. gnavus* and *B. uniformis* was nearly the same (**Figure 5E**). ELISA was performed to evaluate the IL-18 protein released by the Caco-2 cells, which revealed more IL-18 protein in the *B. uniformis* group (**Figure 5F**). Considering that the expression of the IL-18 protein was associated with the cell count and cell density, liner regression was used to adjust the IL-18 protein based on the cell viability and the qRT-PCR (*y* = 197.94 + 307.82 × Cell viability + 7.26 × qRT-PCR, *p* < 0.001) in the healthy model, which showed that the *R. gnavus* group released more IL-18 protein than the *B. uniformis* group. In the inflammatory model induced by 5% DSS, liner regression was also performed (*y* = −958.32 + 5089.52 × Cell viability + 3.20 × qRT-PCR, *p* = 0.003), which obtained similar results (**Figure 5G**). The results of these experiments imply that the supernatant of *R. gnavus* inhibits the growth of the Caco-2 cells and induces the production of IL-18 compared with that of *B. uniformis*. Similar results of the supernatant interfering with the Caco-2 cells for 24 h are displayed in the Supplementary Material (**Supplementary Figures S5A–E**).

## Discussion

This study represents one of the first efforts to discover the AS-associated changes in the human gut microbiome and metabolome in an integrated multi-omics framework, in addition to confirming the effect of TNFi on the microbiome and metabolome with data from a cohort study. TNFi treatment effectively restored the BASDAI, ASDAS, and BASFI scores from high disease activity to low disease activity, while the microbiota and metabolome recovered to a level close to that of the HCs.

For the microbiome, our results presented a difference in the gut microbiota between patients with AS and healthy controls, which is consistent with previous studies showing similar patterns of bacterial phylum distribution in AS, characterized by an increase in Firmicutes and a decrease in Bacteroidetes (4). Utilizing the Wilcoxon rank-sum test and the FDR, we uncovered *R. gnavus* as a pathogenic bacterium and *B. uniformis* as a probiotic. Our results are consistent with those of other studies showing that *R. gnavus* (which belongs to the phylum Firmicutes) played a pathogenic role in several autoimmune diseases (5, 20, 21), while *B. uniformis* (which belongs to Bacteroidetes) was a potential probiotic protecting against metabolic disorders (33). The research findings presented here are consistent with previous studies, establishing the foundation for our forthcoming groundbreaking research. For the first time, a prospective cohort study was conducted to confirm the impact of TNFi on the microbiota in AS. In this cohort study, it was confirmed that TNFi restores the general structure of the gut microbiota in AS to a level similar to that in HCs. Furthermore, it was demonstrated that the quantity of *R. gnavus* recovered to the level of HCs, while the quantity of *B. uniformis* recovered to a level close to that of HCs. Therefore, TNFi could partially alter the gut microbiota structure in patients with AS.

With regard to the metabolome, we were the first to present an untargeted gut metabolome in AS. We discovered differential metabolites between patients with AS and healthy controls using OPLS-DA. In this study, we also validated the impact of TNFi on the metabolome from the aspects of subclass and enrichment pathways. In the subclasses of metabolites, most of the metabolites were not changed by TNFi treatment. Among the changed metabolites, the linoleic acids were previously found downregulated in AS and were associated with cartilage and bone destruction (34). Purine metabolites were found to accelerate the growth of chondrocytes and ectopic new bone formation through PKA/CREB signaling in AS (35). Another study found that serum uric acid, a metabolite of purine, is related to the progression of sacroiliitis in AS (36). This metabolome study found that TNFi therapy restored the subclasses of linoleic acids and purine metabolites. Among the subclasses of unchanged metabolites, bile acid in colon was reported to be influenced by both dietary and microbial factors, act as important hormones that regulate host cholesterol metabolism, and modulate the Treg cells expressing the transcription factor RORγ. Restoration of the intestinal bile acid pool increases the colonic RORγ^+^ Treg counts and ameliorates the host’s susceptibility to inflammatory colitis (37). However, in this study, TNFi therapy was not able to restore the bile acid subclass. From the subclass of metabolites, we can conclude that TNFi partially restores the inflammation-related metabolites. Pathway enrichment analysis further confirmed the change of metabolites from the aspect of enriched metabolic pathways. For instance, the same result was confirmed by the enriched pathways that bile secretion, cholesterol metabolism, primary bile acid biosynthesis, and secondary bile acid biosynthesis remained similar in the Post–HC comparison group to that in the Pre–HC comparison group. TNFi therapy was not able to restore the bile metabolism. The enriched pathways in HCs, such as vitamin digestion and absorption; alanine, aspartate, and glutamate metabolism; and arginine biosynthesis, remained the same in both the Pre–HC and Post–HC comparison groups. Alanine and glutamate metabolism was reported in a serum-targeted metabolomics analysis to be related to inflammation in AS (38). In summary, TNFi could only partially restore some of the inflammatory-related metabolome in AS. This result implies that TNFi cannot change the inflammatory progression radically. These unmodifiable metabolites might contribute to the mechanism of TNFi resistance (39). We expect that exploration of the microbe–metabolite association through multi-omics research will open novel treatment avenues other than TNFi.

This study concurrently examined both the gut microbiota and intestinal metabolites, providing the opportunity to conduct a correlation analysis between them. This is also one of the highlights of our research, which allowed, for the first time in the context of AS, exploring the metabolites associated with potential pathogenic bacteria and beneficial probiotics. The association analysis between the microbiome and the metabolome revealed that *R. gnavus* is negatively associated with oxypurinol and that *B. uniformis* is positively associated with biotin. Oxypurinol, an isostere of xanthine, is a derivative of purine, originating from the body’s endogenous purine metabolism, exogenous intake through food, and as a metabolic product of the drug allopurinol. Purines and purine derivatives were in the top 20 clusters in the Pre group and were altered in the Post group. Xanthine was associated with one of the top 20 pathways, i.e., caffeine metabolism, according to the KEGG database. The urinary levels of oxypurinol in newborns within the first month are in the range 19–37 μmol/mmol creatinine (40); in adults above the age of 18, this ranges from 5.1 to 29.3 μmol/mmol creatinine (41). This reflects a gradual decrease in oxypurinol levels from newborns to adults. In contrast, the quantity of *R. gnavus* in the gut increases gradually from infancy to adulthood, with an increase observed after weaning and the introduction of solid foods (42). This presents a negative correlation between the abundance of *R. gnavus* in the gut and the levels of oxypurinol naturally, and this also supports our multi-omics results. Biotin supports the growth of butyrate-producing bacteria (28, 30). *B. uniformis*, as a butyrate-producing bacterium (29), most likely depends on biotin for proliferation. Interestingly, it has been reported that biotin biosynthesis has been found in a *Bacteroides*-enriched microbiome composition (43). From this, it can be inferred that biotin is a product of *B. uniformis* and stimulates its growth in a positive feedback manner. The above research suggests a positive correlation between the abundance of *B. uniformis* and the levels of biotin, which is similar to our results.

Moreover, the validation experiments further confirmed that oxypurinol inhibited the growth of *R. gnavus*, while biotin promoted the growth of *B. uniformis*. These experiments validated the results from the multi-omics analyses and provided a new perspective for restoring the gut microbiota structure.

To further demonstrate the effect of *R. gnavus* and *B. uniformis* on the intestinal epithelium of the host, we utilized bacterial supernatants to interfere with the Caco-2 cell line, which represented enterocytes. Treatment with the *R. gnavus* or the *B. uniformis* bacterial supernatant increased the tight junction protein ZO-1, in line with studies showing that the *Escherichia coli* Nissle supernatant increased the ZO-1 mRNA to protect the intestinal barrier (44). This suggests that the supernatant does not promote inflammation via tight junction protein effects. Instead, it primarily inhibits cell proliferation while boosting the release of the pro-inflammatory cytokine IL-18. IL-18, as a unique cytokine, participates in the activation and differentiation of various T-cell populations (including Th1, Th2, Th17, and Tregs) (45) and intensifies inflammation by modulating the immune cell responses, as seen in various inflammatory diseases (46). An inflammatory study in patients with AS found an increased IL-18 expression in the ileum (47). The intestinal microbiota could trigger systemic immune responses and arthritis via the IL-18/IL-18R pathway and metabolic products. In this study, it was found that *R. gnavus* produced more IL-18 than *B. uniformis*, potentially causing more inflammation. Inhibiting *R. gnavus* and promoting *B. uniformis* will likely improve the inflammation in the gut and influence the therapy for AS.

A limitation of this study is the lack of an *in vivo* experiment to further validate the effects of pathogenic bacteria and probiotics. Despite this limitation, we have provided a new perspective for AS therapy by presenting the interplay between the gut microbiota, metabolites, TNFi treatment, and the human body. By inhibiting pathogenic bacteria, i.e., *R. gnavus*, and promoting a probiotic, i.e., *B. uniformis*, with the relative metabolites oxypurinol and biotin, respectively, we were able to reduce inflammation by IL-18/IL-18R1 signals.

## Data availability statement

The data presented in this study are deposited in the Genome Sequence Archive (Genomics, Proteomics & Bioinformatics 2021) in National Genomics Data Center (Nucleic Acids Res 2022), China National Center for Bioinformation / Beijing Institute of Genomics, Chinese Academy of Sciences (GSA-Human: HRA006497) that are publicly accessible at https://ngdc.cncb.ac.cn/gsa-human.

## Ethics statement

The studies involving humans were approved by Ethics Committee of Shenzhen Second People’s Hospital. The studies were conducted in accordance with the local legislation and institutional requirements. The participants provided their written informed consent to participate in this study.

## Author contributions

YL: Conceptualization, Data curation, Formal Analysis, Funding acquisition, Investigation, Methodology, Project administration, Resources, Software, Supervision, Validation, Visualization, Writing – original draft, Writing – review & editing. WT: Data curation, Formal Analysis, Methodology, Software, Validation, Writing – review & editing. XL: Formal Analysis, Software, Writing – review & editing. HZ: Methodology, Project administration, Writing – review & editing. YZ: Data curation, Funding acquisition, Investigation, Methodology, Resources, Supervision, Writing – review & editing. MW: Conceptualization, Investigation, Methodology, Resources, Supervision, Writing – review & editing. GY: Methodology, Resources, Software, Supervision, Validation, Writing – review & editing. MY: Conceptualization, Data curation, Formal Analysis, Funding acquisition, Investigation, Methodology, Project administration, Resources, Software, Supervision, Validation, Visualization, Writing – review & editing.

## Supplementary material

Supplementary Figure 1 Details of 170 featured bacterial species that show significant differences between the AS patients and health controls. (adjusted p-value <0.05)

Supplementary Figure 2 Details of 2 differential species between the post and HC groups, and 6 differential species between the post and pre groups. (adjusted p-value <0.05)

Supplementary Figure 3 A, Orthogonal projections to latent structure discriminant analysis (OPLS-DA) between Pre and HC groups. B, OPLS-DA between Post and Pre groups. C, OPLS-DA between Post and HC groups. D, OPLS-DA validated by 200 times response permutation tests in Pre-HC comparison group. E, OPLS-DA validated by 200 times response permutation tests in Post-Pre comparison group. F, OPLS-DA validated by 200 times response permutation tests in Post-HC comparison group.

Supplementary Figure 4 A, Top 20 subclasses of differential metabolites between Post group and Pre group displayed by heatmaps. B, Details of top 20 subclasses of differential metabolites between Post group and Pre group displayed by barcharts. C, Top 20 enrichend pathways of differential metabolites between Post group and Pre group, with GeneRatio represents the ratio of input compound.

Supplementary Figure 5 Validated experiments for effect of species on enterocytes. A, Effect of supernatant from R. gnavus and B. uniformis on proliferation of enterocyte cell model, Caco-2 cell line for 24 hours. B, Effect of supernatant from R. gnavus and B. uniformis on IL-18 mRNA expression of Caco-2 cells for 24 hours. C, Effect of supernatant from R. gnavus and B. uniformis on tight junction protein ZO-1 mRNA expression of Caco-2 cells for 24 hours. D, Effect of supernatant from R. gnavus and B. uniformis on IL-18 protein expression by ELISA for 24 hours. E, Results of IL-18 protein expression adjusted by cell viability and qRT-PCR.
