# Supplementary figures and images for: Gut microbiome and metabolome to discover pathogenic bacteria and probiotics in ankylosing spondylitis
Source: Front Immunol. 2024 Apr 22;15:1369116. doi: 10.3389/fimmu.2024.1369116 (PMC11070502; doi:10.3389/fimmu.2024.1369116)

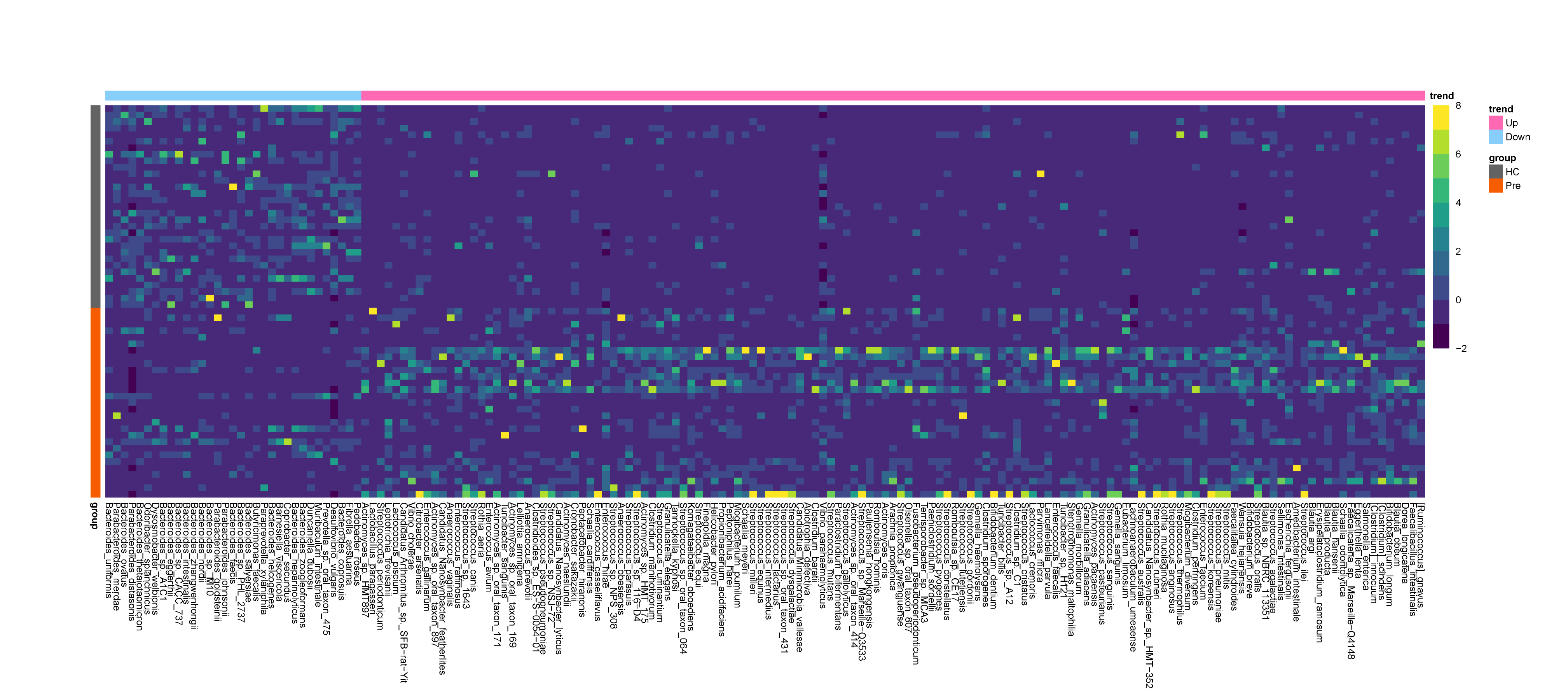

Supplement: Supplementary Figure 1 — Details of 170 featured bacterial species that show significant differences between the AS patients and health controls. (adjusted p-value <0.05) [file Image_1.tif]

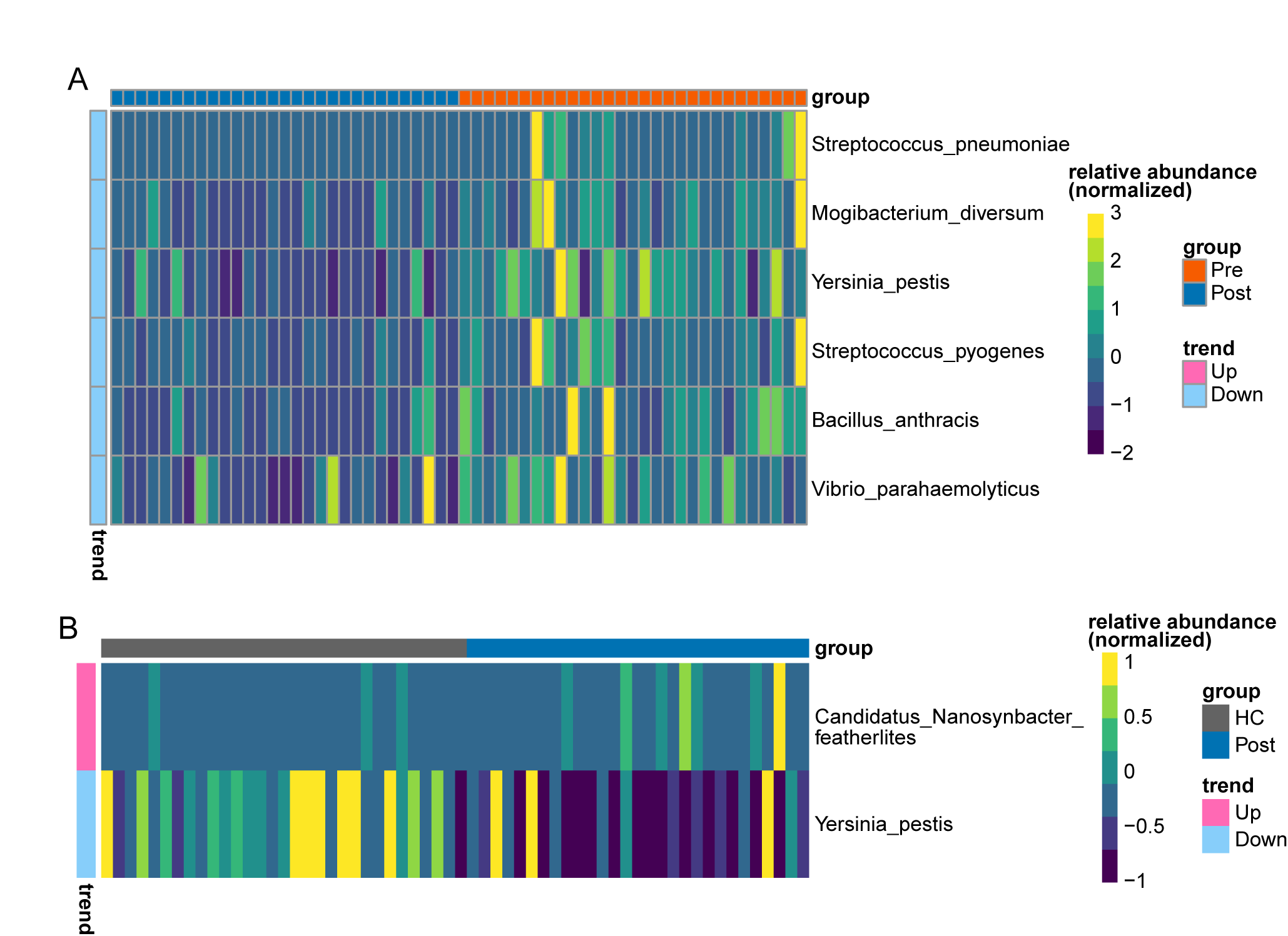

Supplement: Supplementary Figure 2 — Details of 2 differential species between the post and HC groups, and 6 differential species between the post and pre groups. (adjusted p-value <0.05) [file Image_2.tif]

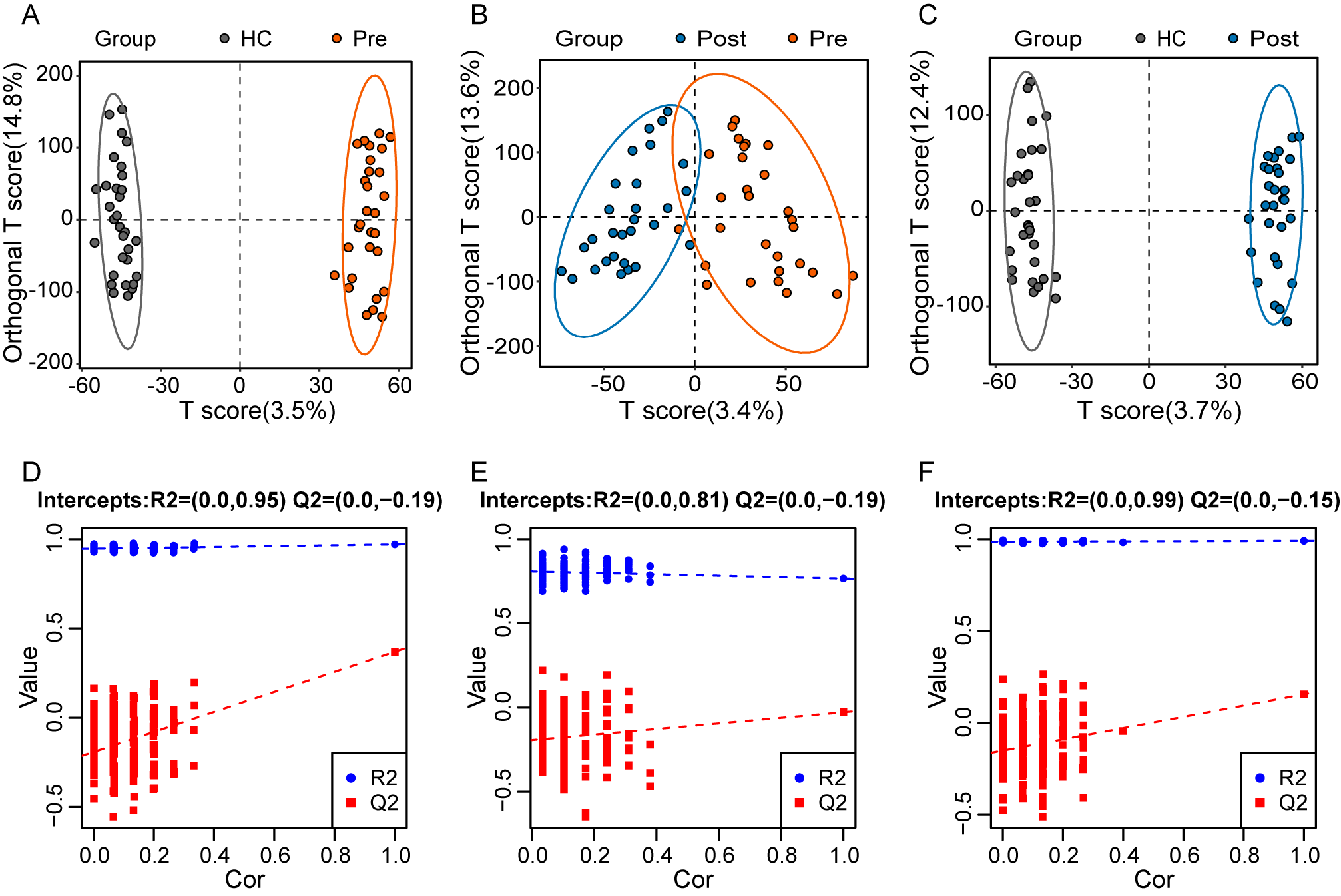

Supplement: Supplementary Figure 3 — A, Orthogonal projections to latent structure discriminant analysis (OPLS-DA) between Pre and HC groups. B, OPLS-DA between Post and Pre groups. C, OPLS-DA between Post and HC groups. D, OPLS-DA validated by 200 times response permutation tests in Pre-HC comparison group. E, OPLS-DA validated by 200 times response permutation tests in Post-Pre comparison group. F, OPLS-DA validated by 200 times response permutation tests in Post-HC comparison group. [file Image_3.tif]

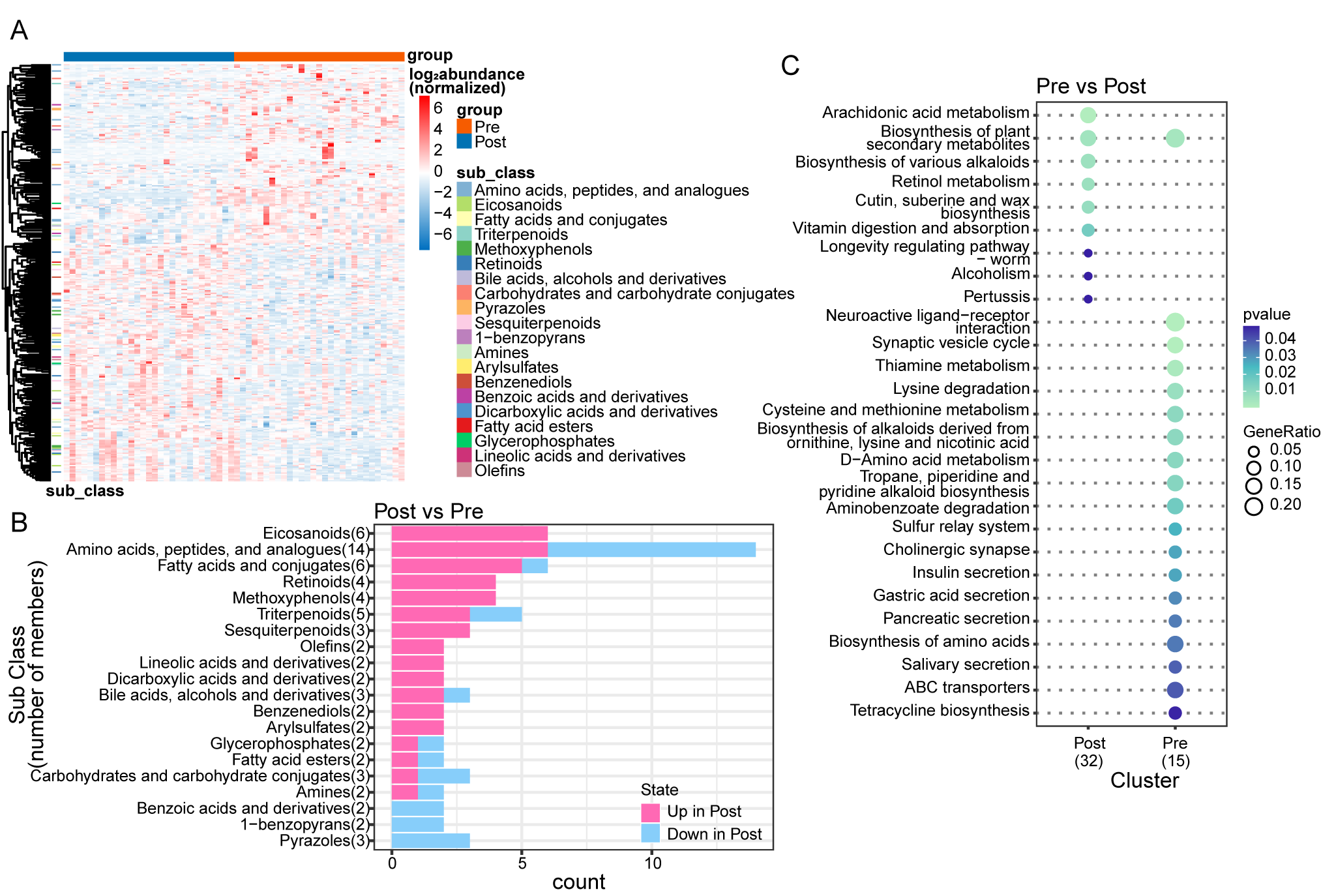

Supplement: Supplementary Figure 4 — A, Top 20 subclasses of differential metabolites between Post group and Pre group displayed by heatmaps. B, Details of top 20 subclasses of differential metabolites between Post group and Pre group displayed by barcharts. C, Top 20 enrichend pathways of differential metabolites between Post group and Pre group, with GeneRatio represents the ratio of input compound. [file Image_4.tif]

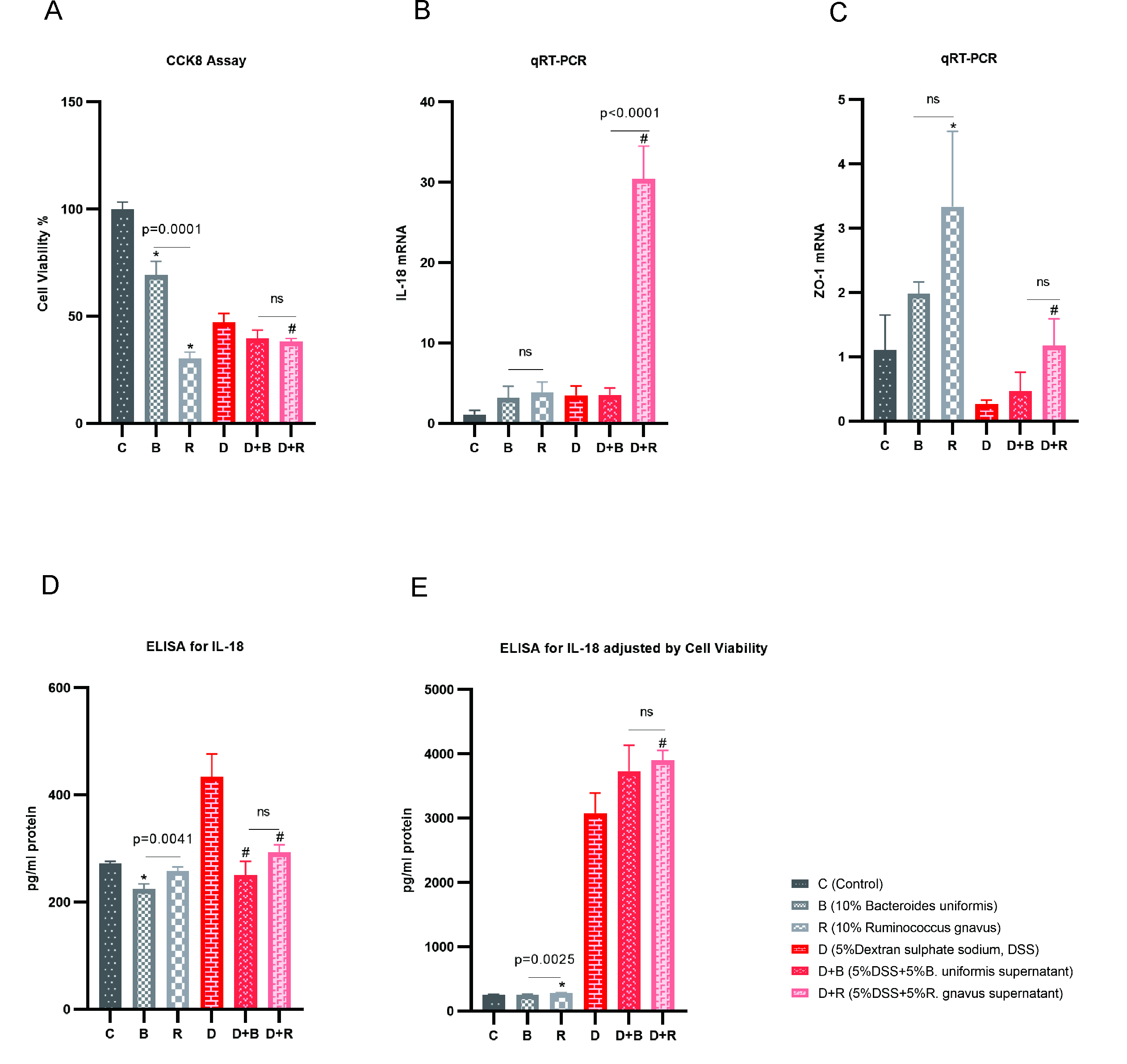

Supplement: Supplementary Figure 5 — Validated experiments for effect of species on enterocytes. A, Effect of supernatant from R. gnavus and B. uniformis on proliferation of enterocyte cell model, Caco-2 cell line for 24 hours. B, Effect of supernatant from R. gnavus and B. uniformis on IL-18 mRNA expression of Caco-2 cells for 24 hours. C, Effect of supernatant from R. gnavus and B. uniformis on tight junction protein ZO-1 mRNA expression of Caco-2 cells for 24 hours. D, Effect of supernatant from R. gnavus and B. uniformis on IL-18 protein expression by ELISA for 24 hours. E, Results of IL-18 protein expression adjusted by cell viability and qRT-PCR. [file Image_5.tif]
