# Supplementary material for: Gut microbiome and metabolome to discover pathogenic bacteria and probiotics in ankylosing spondylitis
Source: Front Immunol. 2024 Apr 22;15:1369116. doi: 10.3389/fimmu.2024.1369116 (PMC11070502; doi:10.3389/fimmu.2024.1369116)
Supplement: Supplementary file 6 [file DataSheet_1.docx]

Supplementary methods

Supplementary subjects’ recruitment

Inclusion criteria for patients: Age ranging from 18 to 60 years old. Diagnosed with1984 modified New York’s criteria for AS or ASAS 2009 classification criteria for axSpA with radiographic images. Patients who have not previously received TNF-α inhibitors or traditional disease-modifying antirheumatic drugs.

Exclusion criteria for patients: Patients with contraindications to TNF-α inhibitors, such as uncontrolled hepatitis B, active tuberculosis, a history of cancer within the past 5 years, sepsis, opportunistic infections, or moderate to severe heart failure, hypertension, diabetes, cardiovascular disease, cerebrovascular disease. Patients who refuse treatment with TNF-α inhibitors. Patients who have received TNF-α inhibitor treatment for less than 1 year. Absolute vegetarians or absolute meat eaters. Patients who have used probiotics within the past 3 months. Patients who have taken antibiotics within the past 3 months. Patients who have had acute gastrointestinal diseases, such as acute gastroenteritis, within the past 3 months.

Inclusion criteria for health controls:

Inclusion criteria: Age between 14 and 60 years. Healthy individuals who have not used prescription drugs.

Exclusion criteria for health controls: History of cancer within the past 5 years. Moderate to severe heart failure, hypertension, diabetes, cardiovascular disease, cerebrovascular disease. Strict vegetarians or strict meat eaters. Use of probiotics within the past 3 months. Use of antibiotics within the past 3 months. Acute gastrointestinal diseases such as acute gastroenteritis within the past 3 months.

Supplementary DNA extraction and metagenomic sequencing

DNA Extraction

Took 100-200mg of the sample and placed it in a centrifuge tube containing grinding beads. Added 1mL of Buffer ATL/PVP-10 and ground the sample using a high-speed homogenizer. Incubated the sample at 65℃ for 20 minutes to facilitate lysis. Next, centrifuged at 14,000g for 5 minutes and transferred the supernatant to a new centrifuge tube. Added 0.6 mL of Buffer PCI and vortexed for 15 seconds. Then, centrifuged at 12,000 rpm for 10 minutes and transferred the supernatant to a deep-well plate. Extracted the genomic DNA from the sample according to the instructions provided with the QIAamp PowerFecal Pro DNA Kit. After the procedure was completed, transferred the DNA solution from the deep-well plate to a 1.5 mL centrifuge tube for storage and used the Qubit instrument to assess DNA quality.

Library Construction and Sequencing

The qualified genomic DNA underwent sonication to achieve a size of 350 bp. Subsequently, end repair was performed, followed by the addition of A base and ligation of DNBSEQ sequencing adapters using the MGIEasy Universal DNA Library Preparation Kit. PCR amplification was carried out on DNA fragments ranging from 300 to 400 bp. Finally, the PCR products were purified using Agencourt AMPure XP Beads, and the sequencing library was assessed using an Agilent 2100 Bioanalyzer. The PCR products were denatured to obtain single-stranded DNA, and a ligation reaction system was prepared. The reaction program was set to generate single-stranded circular products while digesting the remaining linear DNA molecules. Sequencing was performed on the DNBSEQ-2000 sequencing platform, employing a PE 150 sequencing strategy. The single-stranded circular DNA molecules underwent rolling circle replication to form DNA nanoballs (DNBs) that contained multiple copies. The obtained DNBs were loaded onto a high-density DNA nanochip, where they were captured within a mesh of nanopores. Sequencing was conducted using combinatorial Probe-Anchor Synthesis (cPAS) technology.

Filtering for clean data

Raw data with adapter sequences or low-quality sequences was filtered by SOAPnuke v2.2.1 software developed by BGI. We employed the following steps of filtering: 1. Filtered adapter: if the sequencing read matched 50.0% or more of the adapter sequence (with a maximum of 3 base mismatches allowed), the entire read was removed. 2. Filtered read length: if the length of the sequencing read was less than 150 bp, the entire read was discarded. 3. Removed N bases: if the N content in the sequencing read accounted for 1.0% or more of the entire read, the entire read was discarded. 4. Filtered low-quality data: if the bases with a quality value of less than 20 in the sequencing read accounted for 40.0% or more of the entire read, the entire read was discarded. 5. Obtained Clean reads: the output read quality value system was set to Phred+64.

Supplementary metabolites profiling

Metabolite extraction

After defrosting the specimen gradually at 4 °C, measure 25 mg and place it into a 1.5ml Eppendorf tube. Add 800 uL of extraction solution (methanol: acetonitrile: water =2:2:1, v:v:v, pre-cooled at -20 °C) and 10 uL of internal standard. Introduce two small steel balls and subject them to grinding in a tissue grinder (50 Hz, 28/38 BGI Co., Ltd., 400-706-6615 © 2022 BGI All Rights Reserved.) for 5 minutes. Subsequently, perform a 4 °C water bath ultrasound for 10 minutes. Allow the mixture to stand in the refrigerator at 20 °C for 1 hour. Centrifuge at 25000 g for 15 minutes at 4 °C. Take 600 uL of the supernatant after centrifugation and transfer it to a freeze vacuum concentrator for draining. Then, add 600 uL of complex solution (methanol: H2O=1:9, v:v) to dissolve it, vortex the mixture for 1 minute, perform a water bath ultrasound at 4 °C for 10 minutes, and centrifuge at 25000 g for 15 minutes at 4 °C. Place the resulting supernatant in the loading bottle. Extract 50 uL of supernatant from each sample and mix it with synthetic QC samples to evaluate the repeatability and stability of the LC-MS analysis process.

UPLC-MS Analysis

The following chromatographic conditions were employed: Chromatographic separation was conducted using a Waters ACQUITY UPLC BEH C18 column (1.7 μm, 2.1 mm × 100 mm, Waters, USA), and the column temperature was maintained at 45 °C. In the positive mode, the mobile phase comprised 0.1% formic acid (A) and acetonitrile (B), while in the negative mode, the mobile phase consisted of 10 mM ammonium formate (A) and acetonitrile (B). The gradient conditions were as follows: 0-1 min, 2% B; 1-9 min, 2%-98% B; 9-12 min, 98% B; 12-12.1 min, 98% B to 2% B; and 12.1-15 min, 2% B. The flow rate was set at 0.35 mL/min, and the injection volume was 5 μL.

Mass spectrometry conditions were as follows: Q Exactive (Thermo Fisher Scientific, USA) was utilized for primary and secondary data acquisition. The full scan range spanned from 70 to 1050 m/z, with a resolution of 70000. The automatic gain control (AGC) target for MS acquisitions was set to 3e6, and the maximum ion injection time was 100 ms. For subsequent MS/MS fragmentation, the top 3 precursors were selected with a maximum ion injection time of 50 ms and a resolution of 17500. The AGC was set to 1e5. Stepped normalized collision energy was applied at 20, 40, and 60 eV. ESI parameters were configured as follows: Sheath gas flow rate was 40, Aux gas flow rate was 10, and in positive-ion mode, the spray voltage (|KV|) was 3.80, while in negative-ion mode, the spray voltage (|KV|) was 3.20. The capillary temperature was set to 320°C, and the Aux gas heater temperature was maintained at 350°C.
