# Supplementary material for: Gut microbiome and metabolome to discover pathogenic bacteria and probiotics in ankylosing spondylitis
Source: Front Immunol. 2024 Apr 22;15:1369116. doi: 10.3389/fimmu.2024.1369116 (PMC11070502; doi:10.3389/fimmu.2024.1369116)
Supplement: Supplementary file 7 [file Table_1.docx]

Supplementary Table 1 Demographics and disease characteristics of the patients and Health controls

|  | AS patients | Health control | p value |
| --- | --- | --- | --- |
| Number of patients | 29 | 31 |  |
| Age(years) | 33.83 ± 9.48 | 29.94 ± 7.31 | 0.189 |
| Gender(males) | 21 (72.41%) | 23 (74.19%) | 0.876 |

Supplementary Table 2 Paired samples t test for pre-treated and post-treated AS patients

|  | Pre-treated AS patients | Post-treated AS patients | p value |
| --- | --- | --- | --- |
| BASDAI | 3.25 ± 1.58 | 1.72 ± 1.38 | <0.001 |
| ASDAS | 2.49 ± 0.88 | 1.41 ± 0.93 | <0.001 |
| BASFI | 2.26 ± 1.77 | 0.72 ± 1.32 | 0.001 |
